# Amino acid digestibility coefficient values of animal protein meals with dietary protease for broiler chickens

**DOI:** 10.1093/tas/txaa187

**Published:** 2020-10-09

**Authors:** Antonio G Bertechini, Júlio C C de Carvalho, Andressa C Carvalho, Felipe S Dalolio, Jose O B Sorbara

**Affiliations:** 1 Animal Science Department, Federal University of Lavras, Lavras, Minas Gerais, Brazil; 2 Technical Support—DSM Nutritional Products, São Paulo, São Paulo, Brazil

**Keywords:** amino acid ileal digestibility, amino acid total tract digestibility, animal meals, protease

## Abstract

A digestibility experiment was conducted to evaluate the effects of dietary exogenous monocomponent protease on the coefficient of apparent total tract digestibility (ATTD) and coefficient apparent ileal digestibility (AID) of amino acids in meat and bones meal (MBM), poultry byproducts meal (PBPM), and feather meal (FM). A total of 512 Cobb-500 male broiler chickens (aged 14 d) were randomly placed into 64 metabolism cages (8 birds per pen) and were allocated to eight treatments with eight replicates in a semi-controlled environmental room. The experimental diets consisted of the basal diet (corn/SBM) and the replacement of 300 g/kg on a weight basis with MBM, PBPM, or FM. The excreta were collected during 3 d (19 to 21 d), and the ileal digesta (using Celite as an indigestible marker) only 1 d (21 d). The protease contained 75,000 PROT units/g. The use of the enzyme increased (*P <* 0.05) ATTD for alanine, cysteine, glycine, and threonine in the basal diet and AID for the amino acids alanine, cysteine, glycine, lysine, threonine, and valine for the basal diet (vegetable). Regarding meals, there was an increase (*P <* 0.05) in the amino acid digestibility in large part due to the amino acids of MBM (14) and PBPM (9), with only five amino acids for FM. The amino acids glycine and threonine showed increases (*P <* 0.05) in both total and ileal digestibility for all animal meals and for the diet based on corn and soybean meal, which indicates a high specificity of the enzyme for these amino acids. The overall results obtained in this study found satisfactory effectiveness of this exogenous protease. The total collection method was lower by 1.83% of amino acids digestibility than the ileal method.

## INTRODUCTION

The byproducts of rendered animal byproducts from the meat industry such as poultry byproducts meal (PBPM), feathers meal (FM), and meat and bones meal (MBM) are used as protein sources in the diets of birds and contribute to a reduction of production costs for broiler meat and eggs. These sources typically provide 5% to 10% of all amino acids in the diets of the birds.

The PBPM and MBM are considered to have good digestibility (NRC, 1994; Kim et al., 2012), whereas the FM has low digestibility (Kim et al., 2002; Bandegan et al., 2010). Overall, a great variability is found in the nutritional composition of the protein meals. The digestibility of the amino acids (NRC, 1994) is affected by the type of material used (Ravindran and Bryden, 1999) and the industrial processing of these meals (Papadopoulos et al., 1985; Johns et al., 1987; Wang and Parsons, 1998, Shirley and Parsons, 2000; Kim et al., 2002).

Although MBM and PBPM have amino acids with a good digestibility, the use of these nutrients can be improved with the addition of exogenous proteases in the feed. The use of exogenous proteases may reduce the excretion of N into the environment as well as the cost of the poultry feed.

Research has been conducted with monocomponent proteases (Cowieson and Roos, 2016, Cowieson et al., 2019), and the results concerning the improvement in the digestibility of amino acids in feeds are mixed. Bertechini et al. (2009) found significant increases in the digestibility of some amino acids for corn. Carvalho et al. (2009) and Vieira et al. (2016) used a protease as a serine hydrolase with soybean meal and full-fat soybean meal. Additions of the same enzyme improved weight gain (Peek et al., 2009), feed efficiency and digestibility of protein and fat (Freitas et al., 2011), amino acid digestibility in diets based on corn and soybean meal (Maiorka et al., 2009; Peek et al., 2009; Vieira et al., 2009; Angel et al., 2011; Law et al., 2018), and wheat and soybean meal (Freitas et al., 2011; Mohammadigheisar and Kim, 2018). Research with enzyme complexes that contain proteases has also been performed (Zyla et al., 2001; Cowieson and Adeola, 2005; Cowieson and Ravindran, 2008; Stefanello et al., 2016; Singh et al., 2017; Mohammadigheisar and Kim, 2018), and the results do not indicate the real effect of these proteases when associated with other enzymes, which may also have effects on the digestibility of amino acids.

In studies on the digestibility of amino acids from protein sources, the ileal technique (Pertillä et al., 2002; Lemme et al., 2004) has been used as an estimate with adult birds. In most studies, roosters were used to obtain data that are applied to all birds (Parsons et al., 1991), and some are skeptical that such data would be applicable for broilers at their various stages of development (Lemme et al., 2004). The method of the ileal digestibility is accurate in determining the digestibility of amino acids for poultry (Lemme et al., 2004; Bryden et al., 2009). However, the process of determining the ileal digestibility brings some difficulties and raises some questions, such as the proper time for collection after feeding, which ileal segment should be sampled, how to properly stun a bird, the method of slaughter, defining what portion of digesta to collect, the sample quantities needed for analyses (depending on the age of the bird), the indicator type (internal or external) to be used, and the experimental period. The biggest changes occur in the segment of the ileum between Meckel’s diverticulum to the ileocaecal valve. Sebastian et al. (1997) and Bryden et al. (2009) sampled from Meckel’s diverticulum to 40 mm from the ileocaecal valve; Pertillä et al. (2002), Garcia et al. (2007), and Kim and Corzo (2012) up to the ileocaecal valve; Adedokun et al. (2008), Kim et al. (2012), and Cowieson et al. (2019) up to 1 cm from the ileocaecal valve; Bandegan et al. (2010) up to 4 cm from the ileocaecal valve; and Kadim and Moughan (1997) sampled up to 15 to 20 cm of the end of the intestine. There is no standardization for ileal sampling and this may affect the determination of the digestibility of amino acids, as it happens with the microbial action to digest and absorb in this segment (Choct et al., 1999; Gong et al., 2002).

Thus, the objectives of this study were to investigate the effects of dietary monocomponent protease on the coefficient apparent total tract digestibility (ATTD) and the coefficient apparent ileal digestibility (AID) of amino acids in animal protein meals fed to poultry and to compare the total and ileal determinations.

## MATERIALS AND METHODS

Animals were treated humanely and all practices and procedures used in this experiment were in accordance with the *Guide for the Care and Use of Agricultural Animals in Research and Teaching* (FASS, 2010).

### Birds and Housing

A group of male Cobb-500 14-d-old chicks was reared until the beginning of the test in a conventional system (wood shavings for bedding on a concrete floor) in a controlled temperature environment of 32 °C, 29 °C, and 25 °C during weeks 1, 2, and 3, respectively. The birds were acquired from a commercial hatchery, vaccinated against Marek’s disease, and raised with diets based on corn and soybean meal, formulated according to the recommendations by Rostagno et al. (2011). On the 14th day, the birds (512) were starved for 4 h, weighed (405 g ± 5), and randomly allocated into 64 metabolic cages (0.75 × 0.61 × 0.45 m) with 8 birds each. The cage was equipped with a trough-type feeder, a glass-type drinker, and a metal tray for the collection of excreta.

### Experimental Procedures

MBM, PBPM, and FM were the protein meals studied. For each of the protein meals, eight replicates (cages) were randomly selected for each of the test diets and supplied for 14 to 21 d (Table 1).

**Table 1.** Chemical composition of the basal diet and the MBM, PBPM, and FM, as-fed basis (g/kg)^†^

Item	Basal diet	MBM	PBPM	FM
Composition				
Dry matter	898.2	953.1	947.4	917.7
Crude protein	219.1	433.3	585.1	832.8
Crude fiber	56.7	118.8	119.0	50.3
Ash	56.7	377.6	214.9	31.9
Acidity, mg NaOH/g	—	0.68	0.82	0.88
Amino acids				
Alanine	9.5	35.6	37.8	36.0
Arginine	12.5	35.5	41.7	55.7
Aspartic acid	12,9	16.3	27.1	36.1
Cysteine	42.8	3.9	19.2	33.8
Glutamic acid	33.9	27.1	40.8	45.9
Glycine	7.9	55.4	47.5	65.4
Histidine	4.8	5.8	11.4	11.6
Isoleucine	8.3	9.9	24.3	36.2
Leucine	16.2	22.4	42.4	69.2
Lysine	9.9	19.8	27.3	24.0
Methionine	3.9	5.4	10.1	6.7
Phenylalanine	9.3	13.4	24.9	39.8
Proline	11.6	41.2	48.1	51.1
Serine	8.9	15.3	36.9	90.3
Threonine	7.6	12.8	24.3	38.6
Tyrosine	7.4	5.3	16.3	24.6
Valine	10.0	15.8	30.8	60.0

^†^Amino acid analyzed using HPLC methodology.

The corn and soybean meal basal diet used contained 2,980 kcal/kg metabolizable energy, 220 g/kg CP, 11.5 g/kg of digestible lysine, 8.5 g/kg of digestible Met + Cys, 7.6 g/kg of digestible threonine, 10 g/kg of calcium, 5 g/kg of available phosphorus, and 2 g/kg of sodium. The different protein meals replaced 300 g/kg of flour in the diet in this study. The other treatment was protease, which was used in half of the diets according to the schedule presented in Table 2. The protease used was Ronozyme ProAct from DSM Nutritional Products, Basel, Switzerland, EC. 3.4.21. The enzyme was an alkaline serine hydrolase derived from *Nocardiopsis prasina* and produced by *Bacillus licheniformis*, and 1 PROT unit was defined as the amount of enzyme that released 1 μmol of *p*-nitroaniline from 1 μM of substrate [Suc-Ala-Ala-Pro-Phe-*p*-nitroaniline] per minute at pH 9.0 and 37 °C.

**Table 2.** Scheme of diets (as-fed basis)

Test diets	Basal diet (BD), g/kg	Meal, g/kg	Protease, mg/kg
1	1,000	0	0
2	1,000	0	200
3	700 (BD1)	300 (MBM)	0
4	700 (BD 2)	300 (MBM)	200
5	700 (BD 1)	300 (PBPM)	0
6	700 (BD 2)	300 (PBPM)	200
7	700 (BD 1)	300 (FM)	0
8	700 (BD 2)	300 (FM)	200

Feed and water were provided ad libitum and the photoperiod was 23:1 (L:D) h during the study. The birds had 4 d to adapt to the experimental diet, followed by 3 d of collection of excreta (days 19, 20, and 21). The beginning and the end of the excreta sampling were determined by using ferric oxide (10 g/kg) in the feed as a fecal marker. The feed intake and the excreta output were recorded during collection. Samples were collected twice a day, at 8:00 a.m. and at 4:30 p.m., and placed in labeled plastic bags and stored at −5 °C until the end of the test period. At the end of the collection period, the samples were thawed, weighed, and homogenized. Three-hundred grams of aliquots were used for laboratory analyses. A portion of these samples (100 g) was pre-dried in a forced ventilation oven (50 °C) for 72 h. Subsequently, they were weighed, grounded with a “Wiley type” mill with a 0.5-mm sieve, and, then, shipped together with ingredients and experimental diets for dry matter analysis at 105 °C and amino acid analyses. The initial chemical and amino acid compositions of the basal diet and the analyzed ingredients, expressed as as-fed (g/kg), are presented in Table 1.

The basal diet and animal meal samples were analyzed for dry matter, crude protein (CP) (N × 6.25), ether extract, and ash by using the procedures of the Association of Official Analytical Chemists (AOAC, 2000). 

After the period of excreta collection, the birds were starved for 24 h and then had access to food at will for 4 h, with the same diets as in the previous phase, but with 10 g kg− ^1^ of Celite (Scott and Boldaji, 1997) added as an indigestible marker. After this phase, to collect the ileal digesta, four birds were removed per cage and after CO_2_ inhalation they were sacrificed by cervical dislocation. Immediately after slaughter, the ileum was exposed by abdominal incision and the segment between the Meckel’s diverticulum up to 1 cm from the ileocaecal junction was sectioned (Kim et al., 2012). With a light hand pressure on the segment, the contents were collected in plastic containers properly identified and then stored at −4 °C until lyophilization. Samples of excreta and digesta were freeze-dried under vacuum at −40 °C for 72 h, manually macerated, grounded to pass through a 0.5-mm screen, and stored at 4 °C until analysis. The ingredients, digesta, and excreta were analyzed in duplicate by an amino acid (AA)-based methodology using high performance liquid chromatography (HPLC; Shimadzu Corp., Kyoto, Japan; according to White et al., 1986 and Hagen et al., 1989). There was no analysis for the amino acid tryptophan.

### Digestibility Procedures

The coefficient ATTD and AID of amino acids were calculated using the following formula: 

$$\begin{array}{ll} ATTD_{aa}= & \;\;ATTD_{aabasal\;diet}\\ & +\frac{\left(ATTD_{aatestdiet}\;ATTD_{aabasaldiet}\right)}{Meal\;inclusion\;\left(\%\right)} \end{array}$$

$$\begin{array}{l} AID_{aa}=AID_{aabasal\;diet}+\frac{\left(AID_{aatest\;diet}\;AID_{aabasal\;diet}\right)}{Meal\;inclusion\;\left(\%\right)} \end{array}$$

$$\begin{array}{l} AID=\frac{\left[\left(AA/Celite\right)d\;-\;\left(AA/Celite\right)i\right]}{\left(AA/Celite\right)d} \end{array}$$

where ATTD is the coefficient apparent total tract digestibility; AID is the coefficient apparent ileal digestibility; d(AA/Celite) is the dietary ratio of amino acid to Celite; and i(AA/Celite) is the ratio of amino acid to Celite in ileal digesta.

### Statistical Analyses

All data from this experiment were subjected to analysis of variance (ANOVA) with a completely randomized design, using the procedures MIXED of the Statistical Analysis System (SAS; SAS Institute, 2000), version 9.2. The differences in the effects of using the enzyme for each amino acid were determined to be significant (*P* < 0.05) with the Fisher test (*F*-test), according to Steel and Torrie (1980).

## RESULTS

The results for the ATTD and AID of the amino acids in the basal diet, MBM, PBPM, and FM are presented in, Tables 3, 4, 5, and 6 respectively.

**Table 3.** Coefficients of ATTD and AID of amino acids measured according to protease used in the basal diet digestibility study^†^

Amino acid	ATTD^‡^			AID^‡^		
Protease, ppm	0	200	SEM	0	200	SEM
Alanine	0.816^b^	0.826^a^	0.0017	0.816^b^	0.838^a^	0.0024
Arginine	0.867	0.873	0.0035	0.868	0.871	0.0045
Aspartic acid	0.877	0.882	0.0022	0.870	0.889	0.0057
Cysteine	0.794^b^	0.802^a^	0.0018	0.817^b^	0.835^a^	0.0066
Glutamic acid	0.921	0.921	0.0020	0.925	0.928	0.0088
Glycine	0.623^b^	0.656^a^	0.0107	0.645^b^	0.683^a^	0.0067
Histidine	0.878	0.878	0.0022	0.889	0.900	0.0058
Isoleucine	0.865	0.871	0.0013	0.879	0.900	0.0035
Leucine	0.883	0.887	0.0009	0.898	0.889	0.0076
Lysine	0.915	0.921	0.0009	0.910^b^	0.931^a^	0.0035
Methionine	0.919	0.921	0.0034	0.921	0.931	0.0045
Phenylalanine	0.892	0.897	0.0009	0.901	0.898	0.0033
Proline	0.853	0.854	0.0025	0.868	0.860	0.0088
Serine	0.862	0.863	0.0022	0.879	0.899	0.0103
Threonine	0.797^b^	0.817^a^	0.0030	0.817^b^	0.838^a^	0.0088
Tyrosine	0.916	0.919	0.0014	0.909	0.928	0.0067
Valine	0.851	0.860	0.0019	0.862^b^	0.890^a^	0.0060
Total of AA	0.856	0.862	0.0026	0.863	0.876	0.0060

^†^Values are based on means of eight observations.

‡Means in the same row with different superscript letters are significantly different at *P* < 0.05.

**Table 4.** Coefficients of ATTD and AID of amino acids measured according to protease used in the MBM digestibility study^†^

Amino acid	ATTD^‡^			AID^‡^		
Protease, ppm	0	200	SEM	0	200	SEM
Alanine	0.534^b^	0.534^a^	0.0597	0.557^b^	0.598^a^	0.0455
Arginine	0.481^b^	0.550^a^	0.0283	0.506^b^	0.563^a^	0.0265
Aspartic acid	0.667^b^	0.764^a^	0.0420	0.679^b^	0.746^a^	0.0447
Cysteine	0.474^b^	0.516^a^	0.0660	0.485^b^	0.529^a^	0.0555
Glutamic acid	0.640^b^	0.668^a^	0.0460	0.659	0.669	0.0451
Glycine	0.604^b^	0.634^a^	0.0808	0.617^b^	0.648^a^	0.0778
Histidine	0.705^b^	0.735^a^	0.0558	0.707^b^	0.753^a^	0.0671
Isoleucine	0.605	0.616	0.0546	0.614	0.623	0.0456
Leucine	0.699	0.702	0.0986	0.703	0.707	0.0878
Lysine	0.769^b^	0.824^a^	0.0453	0.769^b^	0.827^a^	0.0387
Methionine	0.673^b^	0.688^a^	0.0497	0.646^b^	0.699^a^	0.0467
Phenylalanine	0.675^b^	0.691^a^	0.0299	0.686^b^	0.706^a^	0.0344
Proline	0.529^b^	0.577^a^	0.0908	0.534^b^	0.598^a^	0.0776
Serine	0.645^b^	0.707^a^	0.0438	0.668^b^	0.727^a^	0.0417
Threonine	0.660^b^	0.731^a^	0.0531	0.690^b^	0.758^a^	0.0443
Tyrosine	0.779	0.798	0.0633	0.797	0.801	0.0735
Valine	0.637^b^	0.672^a^	0.0333	0.639^b^	0.683^a^	0.0313
Total AA	0.633^b^	0.673^a^	0.0554	0.643^b^	0.685^a^	0.0514

^†^Values are based on means of eight observations.

^‡^Means in the same row with different superscript letters are significantly different at *P* < 0.05.

**Table 5.** Coefficients of ATTD and AID of amino acids measured according to protease used in the PBPM digestibility study^†^

Amino acid	ATTD^‡^			AID^‡^		
Protease, ppm	0	200	SEM	0	200	SEM
Alanine	0.647^b^	0.661^a^	0.0050	0.656^b^	0.669^a^	0.0049
Arginine	0.823	0.826	0.0074	0.833	0.835	0.0075
Aspartic acid	0.522^b^	0.565	0.0075	0.537^b^	0.580^a^	0.0057
Cysteine	0.707^b^	0.746^a^	0.0077	0.728^b^	0.768^a^	0.0134
Glutamic acid	0.648	0.652	0.0051	0.693	0.696	0.0028
Glycine	0.629^b^	0.651^a^	0.0054	0.639^b^	0.662^a^	0.0054
Histidine	0.702	0.707	0.0070	0.721	0.725	0.0045
Isoleucine	0.735	0.745	0.0068	0.724^b^	0.753^a^	0.0041
Leucine	0.736^b^	0.751^a^	0.0070	0.745^b^	0.760^a^	0.0070
Lysine	0.789	0.796	0.0063	0.798	0.805	0.0034
Methionine	0.811	0.818	0.0089	0.821	0.827	0.0060
Phenylalanine	0.770^b^	0.781^a^	0.0070	0.782^b^	0.813^a^	0.0044
Proline	0.689	0.702	0.0058	0.689	0.702	0.0058
Serine	0.559^b^	0.593^a^	0.0048	0.583^b^	0.617^a^	0.0048
Threonine	0.679^b^	0.760^a^	0.0170	0.696^b^	0.777^a^	0.0112
Tyrosine	0.794^b^	0.815^a^	0.0072	0.812	0.822	0.0074
Valine	0.774	0.787	0.0087	0.787^b^	0.800^a^	0.0035
Total AA	0.707^b^	0.726^a^	0.0073	0.720^b^	0.741^a^	0.0060

^†^Values are based on means of eight observations.

^‡^Means in the same row with different superscript letters are significantly different at *P* < 0.05.

**Table 6.** Coefficients of ATTD and AID of amino acids measured according to protease used in the FM digestibility study^†^

Amino acid	ATTD^‡^			AID^‡^		
Protease, ppm	0	200	SEM	0	200	SEM
Alanine	0.566	0.575	0.0043	0.574	0.583	0.0044
Arginine	0.745	0.747	0.0035	0.752	0.755	0.0035
Aspartic acid	0.546	0.551	0.0036	0.558	0.552	0.0036
Cysteine	0.559^b^	0.621^a^	0.0055	0.564^b^	0.627^a^	0.0056
Glutamic acid	0.633	0.635	0.0041	0.660	0.662	0.0043
Glycine	0.718^b^	0.738^a^	0.0093	0.714^b^	0.733^a^	0.0093
Histidine	0.622	0.628	0.0055	0.640	0.646	0.0057
Isoleucine	0.750	0.754	0.0037	0.759	0.765	0.0037
Leucine	0.695	0.704	0.0046	0.709	0.701	0.0046
Lysine	0.645	0.651	0.0036	0.657	0.664	0.0036
Methionine	0.569^b^	0.588^a^	0.0067	0.593^b^	0.634^a^	0.0069
Phenylalanine	0.750	0.755	0.0042	0.758	0.763	0.0043
Proline	0.412	0.405	0.0072	0.588	0.595	0.0105
Serine	0.837^b^	0.875^a^	0.0136	0.823^b^	0.861^a^	0.0133
Threonine	0.661	0.645	0.0037	0.655^b^	0.671^a^	0.0038
Tyrosine	0.733	0.728	0.0053	0.740	0.745	0.0054
Valine	0.754	0.752	0.0051	0.761	0.758	0.0051
Total of AA	0.658^b^	0.667^a^	0.0055	0.677^b^	0.689^a^	0.0057

^†^Values are based on means of eight observations.

^‡^Means in the same row with different superscript letters are significantly different at *P* < 0.05.

For corn and soybean meal diet, the protease increased (*P* < 0.05) the ATTD and AID only for the amino acids alanine, cysteine, glycine, and threonine, whereas, for lysine and valine, the effect of the protease was observed only in the AID. The major differences in the digestibility of amino acids using the protease were observed for the ileal coefficients. The amino acid glycine was more affected by the use of the protease than other amino acids, and the greatest differences in digestibility were found by either using or not the protease for glycine (5.39% for ATTD and 5.87% for AID).

The effects of the protease on the amino acids of the MBM were more pronounced when compared with the basal diet because a greater number of amino acids were affected by the protease. There was an increase (*P* < 0.05) in the ATTD digestibility coefficients for most of the amino acids, except for isoleucine, leucine, and tyrosine, and glutamic acid, isoleucine, leucine, and tyrosine for AID. The effects of the protease were greater than 10% for the amino acids arginine, aspartic acid, and threonine, and the effects were similar for both ATTD and AID.

The effects of using protease were similar to MBM for ATTD and AID with PBPM (Table 5). There was a significant increase (*P* < 0.05) in the digestibility coefficient for the amino acids alanine, aspartic acid, cysteine, leucine, phenylalanine, serine, threonine, tyrosine (only ATTD), and total amino acids. The increases in the digestibility coefficient were different for each amino acid. The largest effects on coefficients were observed for the amino acids threonine, aspartic acid, serine, and cysteine. The increases in ATTD for these amino acids were 11.97%, 8.22%, 6.08%, and 5.64%, respectively. Similar increments were also observed for AID.

When it comes to FM, there were increases (*P* < 0.05) with the use of the protease in ATTD only for the amino acids cysteine, serine, methionine, and average of AA. For the AID of the amino acids, there was a significant increase (*P* < 0.05) for threonine. Additionally, the increases in AID were approximately 11.13%, 6.79%, 4.62%, and 2.38% for cysteine, methionine, serine, and threonine, respectively.

## DISCUSSION

The analyses of different meals revealed that the initial composition of amino acids was different. The MBM was relatively low in protein and amino acids and had a high concentration of bones, as indicated by the high ash content. The PBPM had average contents of crude protein and amino acids according to the tables of food composition (NRC, 1994; Rostagno et al., 2017) and had high levels of fat and bone ash. For the FM, the crude protein and amino acid contents were high, with small concentrations of fat and bone ash. All protein meals were evaluated for an acidity index and had normal values, which indicated an acceptable quality.

**Figure 1.**
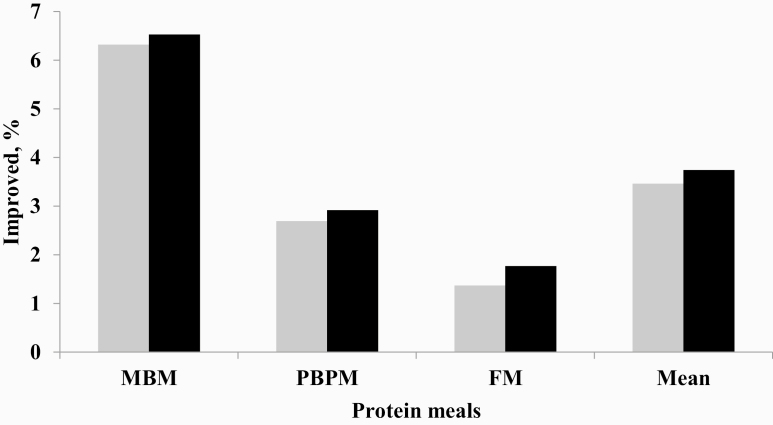
Effect of dietary protease on amino acids digestibility increase (%) of the animal protein meals for broiler diet.

The effects of using this protease on the ATTD as well as the AID of amino acids were different between the vegetable diet and animal meals (Figure 1—significant effect—*P <* 0.05). The enzyme had major effects on the animal protein meals with larger increases in amino acid digestibility for a greater number of amino acids than in the vegetable diet (basal). In the case of the basal diet, there were increases in the digestibility for only four amino acids (alanine, cysteine, glycine, and threonine) for ATTD and for six amino acids for AID (alanine, cysteine, glycine, lysine, threonine, and valine). In another study with a diet based on corn and soybean meal with the same evaluation period and the same amount of the enzyme (200 ppm), Angel et al. (2011) found larger increases in the digestibility of cysteine (4.6% vs. 2.82%), lysine (5.4% vs. 2.32%), and threonine (7.8% vs. 2.57%) in the AID when compared with the results of this work. However, the authors used a basal diet that contained low crude protein, unlike the present study, where the protein level met the requirements of the birds for the period under study. This result suggested that the amplitude of the protease effect depended on the basal diet CP level. With protein-deficient diets, the expected protease effects are greater. The CP level of the basal diet had a significant influence on the AA digestibility study with protease (Rada et al., 2016).

Significant increases occurred in the digestibility (ATTD and AID) of 14 amino acids in the MBM (alanine, arginine, aspartic acid, cysteine, glycine, glutamic acid, histidine, lysine, methionine, phenylalanine, proline, serine, threonine, and valine), 8 amino acids in the PBPM (alanine, aspartic acid, cysteine, leucine, phenylalanine, serine, threonine, and tyrosine) for ATTD, and 9 for AID (alanine, aspartic acid, cysteine, leucine, isoleucine, phenylalanine, serine, threonine, and valine), and 4 amino acids in the FM (cysteine, methionine, serine, and threonine). Only the amino acids serine and threonine increased (*P* < 0.05) in all protein meals, with average increases (ATTD and AID) of 9.19% and 10.25%, respectively. When compared with the results of research with other monocomponent proteases, the enzyme had a greater effect on some amino acids than on others. Angel et al. (2011), in a study with the same enzyme used in this study, showed an enhanced ileal digestibility of serine and threonine by 5.5% and 7.8%, respectively, in diets based on corn and soybean meal for a similar period. In this study, no significant increases were observed (*P >* 0.05) in the ileal digestibility of serine, and the only increase (*P* < 0.05) in the AID was a 2.57% increase in the digestibility of threonine. In another work with the same protease, an increased digestibility of some amino acids in soybean meal (Bertechini et al., 2009), corn (Carvalho et al., 2009), and corn/wheat-based diets (Cowieson et al., 2019) were reported.

For MBM, significant increases (*P* < 0.05) in the digestibility coefficients for a large proportion of the dietary amino acids were found, with the largest increases in the ATTD for arginine (14.29%), aspartic acid (12.9%), threonine (10.67%), serine (9.59%), proline (8.96%), cysteine (8.68%), methionine (7.97%), alanine (7.41%), lysine (7.22%), and valine (5.46%). For the total amino acids, there was an increase (*P* < 0.05) in the ATTD of 6.45%. The increases in the AID were similar to those observed in the ATTD, with minor differences, which resulted in a 6.35% increase for all amino acids with the use of the protease. The AID was higher for the amino arginine (11.5%), proline (11.5%), threonine (10.0%), aspartic acid (10.0%), cysteine (9.1%), serine (8.8%), methionine (8.4%), and lysine (7.5%). These values are higher than those by Pertila et al. (2002) with a similar determination period (21 × 24 d). Moreover, the basal diet used by Pertilla et al. (2002) was semi-purified.

The digestibility coefficients of various amino acids in the PBPM increased (*P* < 0.05) with the use of the protease. The largest increases were observed for the ATTD for the amino acids threonine (11.97%), aspartic acid (8.22%), serine (6.08%), and cysteine (5.65%). For the AID, the same amino acids showed similar increases and the increases were 11.67% for threonine, 7.99% for aspartic acid, 5.85% for serine, and 5.65% for cysteine. The average apparent digestibility for total amino acids increased (*P* < 0.05) by using the protease, and the increases were 2.75% and 2.43% for ATTD and AID, respectively, for PBPM. The digestibility values found in this study were similar to those observed for this meal by Garcia et al. (2007) with roosters.

For the FM, only the amino acids such as cysteine, methionine, and serine had significant increases (*P* < 0.05) in the ATTD, with values of 11.24%, 3.46%, and 4.54%, respectively, with the use of the protease. According to the coefficient for AID, there was also an increase (*P* < 0.05) for threonine. The increases observed were 10.8%, 6.9%, 4.6%, and 2.4% for cysteine, methionine, serine, and threonine, respectively. When total amino acids were analyzed, the use of protease significantly increased the digestibility (*P* < 0.05) by an average of 9.7 for the AID.

Previously, this meal had a low reported digestibility of amino acids (Kim et al., 2002; Bandegan et al., 2010). However, the results found in this study indicated a better quality of the meal due to the higher coefficients observed.

When comparing the effects of the protease on the digestibility of amino acids in the three protein meals studied, a greater number of amino acids in the MBM were affected, followed by PBPM and with a lower effect on FM amino acids. The composition of the meal provided an explanation for a better overall digestibility of amino acids in the MBM in comparison to the other two meals. Large variations in amino acid digestibility due to the differences in composition and processing were noted in other studies of amino acid digestibility with these meals (Papadoupoulos et al., 1985; Latshaw, 1990; Wang and Parsons, 1998; Shirley and Parsons, 2000; Kim et al., 2002).

The protease monocomponent that was used in this study had a higher affinity for some amino acids, regardless of the source of protein. With the enzyme, the total and ileal digestibility of the amino acids such as cysteine, glycine, and threonine increased (*P* < 0.05). Considering only MBM, in addition to these amino acids, the digestibility of serine was also significantly increased (*P* < 0.05) with the protease. These meals have large concentrations of this amino acid for the favorable action of this enzyme.

The use of this protease can improve the utilization of amino acids in the diets of birds and can contribute to a reduced need for amino acids and to reduced levels of dietary crude protein and subsequent N excretion into the environment. By comparing the ATTD and AID, for most sources of protein and amino acids, the values obtained for the ileal digestibility were higher than the ATTD. However, a correlation with the total determinations was observed. The largest difference was obtained for the comparison of animal meal and vegetable diets. Thus, the ileal digestibility values were more accurate, as indicated by Payne et al. (1968), Ravindran et al. (1999), and Lemme et al. (2004), among others, once the effects of microbial activity of the cecum and colon were absent. Moreover, the differences between the treatments in this study ranged on average 1.83% (corn and soybean meal = 1.28%; MBM = 1.70%; PBPM = 1.72%, and FM = 2.60%), and the treatments did not significantly change the digestibility coefficients. Schøyen et al. (2007) found the differences to be also small (<2%) in the studies of protein sources for broilers. The effects of the cecum and colon were significant only in studies when this compartment was allowed to reach sources of energy (carbohydrates) in which the microbiota might develop and modify the amino acid profile of excreta (Parsons et al., 1982). For the ileal determinations, there is a need to cull birds properly (stress) and to use an indigestible fecal marker (errors in the determination) and due to the greater number of calculations and analyses, it can be inferred that the evaluation of sources for protein digestibility using total fecal collection provided satisfactory results.

An entire scientific movement is in favor of ileal digestibility as being the most suitable to evaluate the digestibility of amino acids in the diets of birds. However, the applicability of the values of ileal digestible amino acids is still questionable in practice. The digesta samples in the ileal method represent only one collection point (Bedford and Cowieson, 2019), unlike the collection of excreta that represents a phase of feed intake and thus can better represent the amino acid digestibility.

In conclusion, the present study found positive effects of the protease to enhance the amino acid digestibility of amino acids from MBM, PBPM, and FM to the diets of broilers. The use of this protease could contribute to a reduction in crude protein levels and amino acids required in the diets of broilers and nitrogen in the environment. The AID was on average 1.83% more than the ATTD.
